# Ileocecal Intussusception With Lipoma as a Lead Point Leading to Small Bowel Obstruction in an Elderly Male: A Case Report

**DOI:** 10.7759/cureus.28919

**Published:** 2022-09-08

**Authors:** Syed Faqeer Hussain Bokhari, Khizer Yaseen, Shaun Abid, Rimsha R Vohra, Samar Sajid

**Affiliations:** 1 Medicine and Surgery, Mayo Hospital, Lahore, PAK; 2 Internal Medicine, Hamdard College of Medicine & Dentistry, Karachi, PAK; 3 Internal Medicine, Allama Iqbal Medical College, Lahore, PAK; 4 Internal Medicine, Dow University of Health Sciences, Karachi, PAK; 5 Medicine, Dow University of Health Sciences, Karachi, PAK

**Keywords:** surgical resection, benign tumor, polypoidal lipoma, intraluminal mass, ileocecal intussusception, submucosal lipoma, bowel obstruction, colonic lipoma, intussusception

## Abstract

Intussusception with a lipoma as a lead point and associated bowel obstruction is very uncommon in adults. The patient presents with abdominal pain and intermittent or sudden intestinal obstruction requiring surgical intervention. We report the case of a 68-year-old male who presented with complaints of severe abdominal pain, nausea, vomiting, and constipation. A CT scan revealed ileocecal intussusception with a lipoma as a lead point. Evidence of small bowel obstruction and ascites was also noted on imaging. The patient underwent an ileocecal resection followed by an ileocolic side-to-side anastomosis. Pathological examination of the specimen revealed two adjacent submucosal lipomas with focal mucosal ischemic hemorrhagic changes of the large distal lipoma. We present this case owing to its rarity and believe that it will serve to broaden the horizon of research regarding intussusceptions secondary to submucosal lipomas.

## Introduction

Intussusception is defined as the telescoping of a proximal segment of the intestine into the distal segment. It was first described by Barbette of Amsterdam in 1674 [1]. Intussusception is more common in infants under three years of age and is usually idiopathic. It is a rare condition in adults, often resulting from an underlying pathology [2,3]. Intussusception in adults accounts for only 3% of all intussusceptions and only 1% of these may result in intestinal obstruction [4]. The possible causes of intussusception in adults include some gastrointestinal malignancy, diverticulum, or any polyp of adequate size, which operate as a lead point and predispose to intussusception. Intussusception with a submucosal lipoma as a lead point usually presents with symptoms of obstruction in adults such as abdominal pain, nausea, vomiting, and changes in bowel habits [5]. It frequently has an acute nature if there is associated intestinal obstruction and the patient generally presents in an emergency setting. In the absence of obstruction, there may be no symptoms and the intussusception may be diagnosed incidentally on colonoscopy or radiological imaging. Surgical intervention is especially required in cases involving intestinal obstruction and necrosis [6].

## Case presentation

A 68-year-old male presented to the emergency department complaining of severe abdominal pain, nausea, vomiting, and constipation. The patient had a past medical history of hypertension, emphysema, and chronic bronchitis. He was a chronic smoker (16 pack years) with no past allergies. Physical examination showed abdominal distention with generalized tenderness, particularly in the right iliac fossa. Rigidity or rebound tenderness was absent. On administration, his blood pressure was 110/60 mmHg, pulse rate of 90 bpm, respiratory rate of 16 breaths per minute, oxygen saturation of 89% at room air, and fever of 36.7^0^C. Complete blood count (CBC), liver function tests (LFTs), and renal function tests (RFTs) were normal.

**Figure 1:**
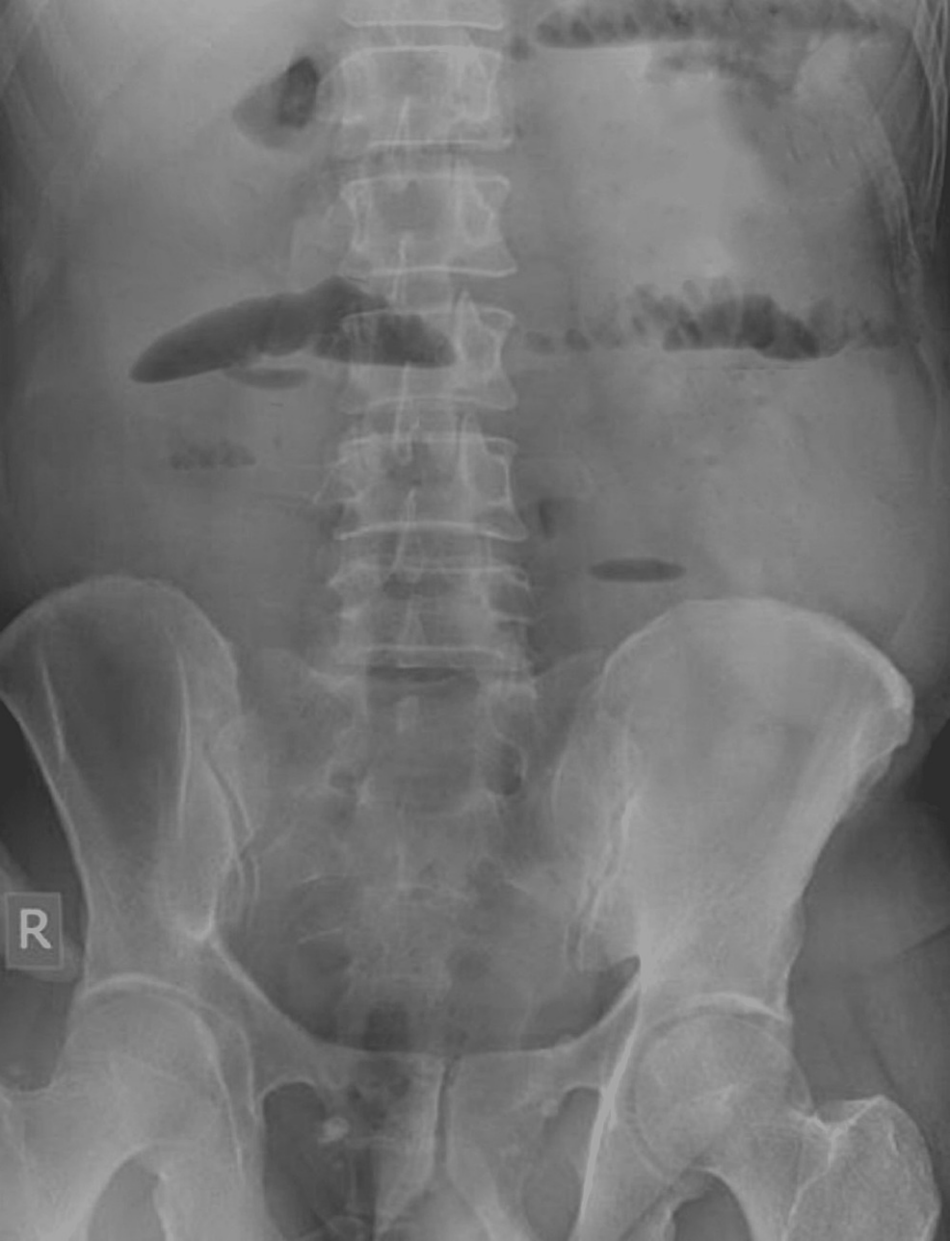
X-ray of abdomen indicating intestinal obstruction.

**Figure 2:**
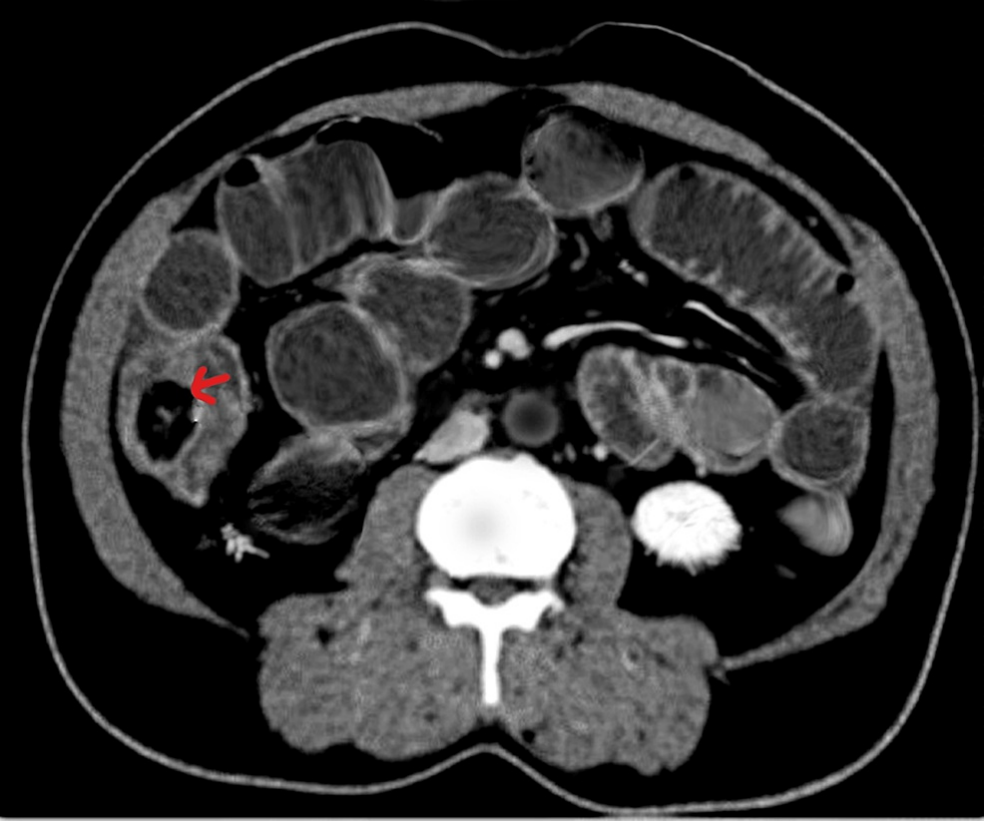
Axial CT scan of abdomen showing an intraluminal lesion in the ileocecal junction (red arrow) and dilated proximal small bowel loops.

An x-ray of the abdomen showed multiple air-fluid levels suggestive of bowel obstruction (Figure 1). Ultrasonography did not show any specific signs due to bowel gas. Computed tomography (CT) scan of the abdomen and pelvis was done, which confirmed small bowel obstruction. A well-circumscribed, intraluminal mass with radiolucent center was observed at the ileocecal junction measuring 2.5 × 1.5 cm (Figure 2). The terminal ileum had entered the cecum, thereby indicating ileocecal intussusception. Mild ascites was also observed. Initially a tumor obstructing the terminal ileum was diagnosed. Ciprofloxacin 500 mg, metronidazole 500 mg, omeprazole 40 mg, and paracetamol 1 g/100 ml was administered to the patient.

Emergency laparotomy was performed and the diagnosis of ileocecal intussusception was confirmed. The caecum, appendix, and 70 mm of terminal ileum were resected. Two adjacent polyps within the terminal ileum serving as lead points were revealed during exploration of the resected mass. Both were yellow, lobular, and pedunculated, indicating a lipoma. The distal one was larger, at a distance of 5.3 cm from the ileocecal valve, and was protruding through the ileocecal valve into the cecum. The resected mass was sent for histopathological analysis. Side-to-side anastomosis of the ileum to the ascending colon was performed. The surgery was successful and the patient was hemodynamically stable and afebrile. 

The post-surgical histopathological examination showed a large distal polypoidal lipoma of terminal ileum lined by ulcerative bowel mucosa (6 cm in the greatest dimension) and an adjacent small proximal polypoidal lipoma of terminal ileum lined by ulcerative bowel mucosa (2 cm in the greatest dimension). Focal mucosal ischemic hemorrhagic changes of large distal lipoma were observed. The proximal and distal resection margins were unremarkable. A small paracolic lymph node was also revealed, which was unremarkable, incidental, pericolic, and benign.

## Discussion

Intussusception is a relatively rare condition in adults as compared to children. Depending upon its location, it may be classified as enteroenteric, colocolic, ileocolic, or ileocecal [3]. Contrary to its idiopathic etiology in children, 90% of the cases in adults are secondary to an underlying etiology. The most common causes include benign neoplasms, adhesions, Meckel's diverticulum, lymphoid hyperplasia, and adenitis. Intestinal lipomas are quite rare and have a reported frequency of 0.15-4.4%, with the majority developing in the large intestine [7]. Lipomas are uncommon benign tumors that may develop in the bowel and when greater than 2 cm in size may lead to intestinal obstruction and intussusception. As compared to children, the clinical presentation in adults is usually acute and is not readily diagnosed based on clinical signs and symptoms [8,9]. The most common presenting complaints include generalized abdominal pain, abdominal distention, nausea and vomiting, constipation, and changes in bowel habits [10]. Ultrasonography is a cost-effective, feasible, and non-invasive technique to detect submucosal lipomas. They are visualized as an oval or round mass presenting with a classic doughnut shape sign [11]. However, due to bowel gases, which are almost always associated with abdominal obstruction, ultrasonography may not be able to detect the lipoma as it is difficult to visualize in such circumstances [12]. CT scan is an ideal modality for establishing a confirmed case of an intestinal lipoma. It not only pinpoints the site and size of the lipoma but also shows the associated complications such as intestinal obstruction, bowel gases, perforations, hemorrhages, lymphadenopathy, etc. [13].

In adults with intussusception, nonoperative reduction with barium or air is not the preferred therapy as it is in children due to an underlying pathological lead point or predisposing disease [14]. As a result, surgical resection is the best course of action for intussusception in adults [15]. The risks associated with reducing an intussusceptive bowel include intraluminal seeding and venous tumor dissemination, perforation and seeding of microorganisms and tumor cells into the peritoneal cavity, as well as an increased risk of anastomotic complications of manipulated friable and edematous bowel [16]. Another possible method of care is the endoscopic removal of lipomatous polyps and the laparoscopic removal of benign small intestinal tumors producing intussusception.

## Conclusions

Ileocecal intussusceptions secondary to lipoma as a lead point are quite rare in adults. They may lead to intestinal obstruction, which results in a medical emergency. This condition presents with abdominal pain and symptoms of bowel obstruction but cannot be diagnosed on clinical examination alone. Despite the fact that intussusception itself has a favorable prognosis, the key prognostic factor influencing the course of disease is the type of causing lesion. Imaging modalities such as ultrasonography and CT scan are used to establish the final diagnosis. Ultrasonography may be inconclusive due to the presence of bowel gases. Surgical intervention is generally required for treating this condition. Intussusception is confirmed on exploration during surgery. Lipoma is confirmed on post-surgical histopathological examination.
